# Rice bran extract: an inexpensive nitrogen source for the production of 2G ethanol from sugarcane bagasse hydrolysate

**DOI:** 10.1007/s13205-012-0098-9

**Published:** 2012-10-20

**Authors:** Thais S. S. Milessi, Felipe A. F. Antunes, Anuj K. Chandel, Silvio S. Silva

**Affiliations:** Departamento de Biotecnologia, Universidade de São Paulo, Escola de Engenharia de Lorena, Estrada municipal do Campinho s/n, P.O. Box 116, Lorena, SP 12810-030 Brazil

**Keywords:** Rice bran extract, Bioethanol, *Scheffersomyces stipitis* NRRL Y-7124, Sugarcane bagasse, Nitrogen source

## Abstract

Selection of the raw material and its efficient utilization are the critical factors in economization of second generation (2G) ethanol production. Fermentation of the released sugars into ethanol by a suitable ethanol producing microorganism using cheap media ingredients is the cornerstone of the overall process. This study evaluated the potential of rice bran extract (RBE) as a cheap nitrogen source for the production of 2G ethanol by *Scheffersomyces* (*Pichia*) *stipitis* NRRL Y-7124 using sugarcane bagasse (SB) hemicellulosic hydrolysate. Dilute acid hydrolysis of SB showed 12.45 g/l of xylose and 0.67 g/l of glucose along with inhibitors. It was concentrated by vacuum evaporation and submitted to sequential detoxification (neutralization by calcium hydroxide and charcoal adsorption). The detoxified hydrolysate revealed the removal of furfural (81 %) and 5-hydroxymethylfurfural (61 %) leading to the final concentration of glucose (1.69 g/l) and xylose (33.03 g/l). *S. stipitis* was grown in three different fermentation media composed of detoxified hydrolysate as carbon source supplemented with varying nitrogen sources i.e. medium #1 (RBE + ammonium sulfate + calcium chloride), medium #2 (yeast extract + peptone) and medium #3 (yeast extract + peptone + malt extract). Medium #1 showed maximum ethanol production (8.6 g/l, yield 0.22 g/g) followed by medium #2 (8.1 g/l, yield 0.19 g/g) and medium #3 (7.4 g/l, yield 0.18 g/g).

## Introduction

Second generation (2G) ethanol derived from lignocellulosic materials is a sustainable alternative to fossil fuels and provides unique environmental, economic, and strategic benefits (Dale and Ong 2012). Lignocellulosic materials are the renewable, sustainable and near-term feedstock used to produce the 2G ethanol. Among them, sugarcane bagasse (SB) is one of the most promising feedstock for ethanol production in countries like Brazil, India and China due to the large availability (279 million metric ton production in 2011) and does not compete with food/feed demands (Chandel et al. 2012). Furthermore, the high-carbohydrate composition in the SB (circa 75 %) made it excellent raw material for its implication in fermentation reactions as carbon source by microorganisms (Soccol et al. 2010; Macrelli et al. 2012).

To obtain the desired ethanol production efficiency from SB, hemicellulose depolymerization is required. Dilute acid hydrolysis is an effective and well established process to depolymerize the hemicellulose into fermentable sugars leaving cellulignin in the substrate (Gírio et al. 2010). However, some undesired products so called inhibitors like furans, phenolics and weak acids are also generated during this process which can be eliminated by sequential detoxification methods such as overliming and activated charcoal treatment to obtain the competitive ethanol yield (Chandel et al. 2007).

Of the various xylose fermenting yeasts, *Scheffersomyces stipitis* is the promising one for ethanol production due to its ability to ferment a wide variety of sugars present in the hemicellulose hydrolysates, non-requirement of vitamins or amino acid supplements for growth and no production of xylitol under limited oxygen supply (Agbogbo and Coward-Kelly 2008; Jeffries and Jin 2004; Canilha et al. 2010). Previously known as *Pichia stipitis*, this yeast had its gender modified to *Scheffersomyces* after phylogenetic analysis (Kurtzman and Suzuki 2010). The use of low-cost fermentation additives would most likely decrease ethanol production costs. After carbon source, nitrogen is the most important component of the fermentation media and is required by microorganisms for metabolite production at acceptable yield and productivity levels. Routinely used chemicals such as yeast extract and peptone as nitrogen source are expensive and contribute almost 50 % of the cost of ethanol production (Ananda et al. 2011). Rice bran extract (RBE) can be used as a nitrogen source and holds the potential to replace the costly nitrogen sources simultaneously reducing the production cost of fermentation-based processes (Arruda 2007; Ananda et al. 2011). Rice bran, a byproduct produced during the steps associated with brown rice polishing into white rice grains, contains nutrients that could be used as a nitrogen supplement to microorganisms (Moro et al. 2004). Considering the importance of RBE to reduce the ethanol production cost and its future viability at large scale operations, this study aims to explore the potential of RBE as a nitrogen source for ethanol production from sugarcane bagasse hemicellulosic hydrolysate using *S. stipitis*.

## Materials and methods

### Raw material and hydrolysis

Sugarcane bagasse was provided by Usina São Francisco located at Sertãozinho/São Paulo, Brazil. It was used as received from the factory. It was acid-hydrolyzed in a hydrolysis reactor with a capacity of 100 l located at the Department of Biotechnology of Engineering School of Lorena (EEL)-USP, Lorena, Brazil. The bagasse was percolated with H_2_SO_4_ (98 % of purity) at a ratio of 100 mg H_2_SO_4_/g of dry material for 20 min at 121 °C, using a ratio of 1/10 of bagasse mass and volume of H_2_SO_4_ solution (Pessoa et al. 1997). After hydrolysis, the reaction mixture was cooled, subsequently recovered and was stored at 4 °C.

### Concentration of acid hydrolysate by vacuum evaporation and detoxification

The hydrolysate was concentrated under reduced pressure by a concentrator of capacity of 32 l at 70 °C to obtain the xylose concentration of 50 g/l. This concentrated hydrolysate was detoxified according to the method of Alves et al. (1998) that involves three stages: raising the pH to 7.0 with calcium oxide; reduction of pH to 5.5 with phosphoric acid (85 % of purity) and addition of activated charcoal (2.4 % w/v) for 1 h at 200 rpm and 30 °C. After each stage, the hydrolysate was filtered under vacuum and after the last stage it was autoclaved at 0.5 atm (110 °C) for 15 min and was used in the fermentation reactions.

### Microorganism and inoculum preparation

*S. stipitis* NRRL Y-7124 was used for the fermentation of detoxified hemicellulosic hydrolysates. Strains were maintained on YPD (yeast extract, peptone, dextrose and agar) plates and stored at 4 °C.

The inoculum was prepared by transferring a loopful of strains from the slant into 125 ml Erlenmeyer flasks containing 50 ml of synthetic medium composed by 30.0 g/l of xylose, 3.0 g/l of yeast extract and 5.0 g/l of peptone (Ferreira et al. 2011). The flasks were incubated in a rotatory shaker (Innova 4000 Incubator Shaker, New Brunswick Scientific, Enfield, CT, USA) at 200 rpm and 30 °C for 24 h. Following 24 h growth, broth was centrifuged (2,777.1×*g* for 10 min) and inoculum was prepared corresponding to 1.0 g/l cells (d. wt). Inoculum was aseptically transferred into detoxified acid hydrolysate (50 ml) supplemented with medium ingredients.

### Rice bran extracts (RBE) preparation

The rice bran was kindly gifted by Professor Ismael Maciel de Mancilha from EEL to USP, Lorena. It was autohydrolyzed in a ratio of 200 g of rice bran to 1 l of distilled water. The mixture was autoclaved for 15 min at 110 °C. After cooling, the mixture was centrifuged (C-5000, Damon/IEC Division, Needham Heights, MA) at 1,234.3×*g* at ambient temperature for 30 min under aseptic conditions. The supernatant (RBE) was transferred to a sterilized flask aseptically and was used as a nitrogen source in place of yeast extract or peptone (Arruda 2007).

### Fermentation medium and conditions

Three different fermentation media (medium#1, #2, and #3) were accessed for ethanol production by *S. stipitis* NRRL Y-7124. Medium#1 was composed of hemicellulosic hydrolysate supplemented with RBE (10 % v/v) as nitrogen source, CaCl_2_ (0.1 g/l) and (NH_4_)_2_SO_4_ (2.0 g/l) (Milessi et al. 2011). Medium#2 was composed of hemicellulosic hydrolysate supplemented with yeast extract (3.0 g/l) and peptone (5.0 g/l) (Canilha et al. 2010). In medium #3, hydrolysate was supplemented with yeast extract (3.0 g/l), peptone (5.0 g/l) and malt extract (5.0 g/l) (Canilha et al. 2010).

All the fermentation assays were carried out in 125 ml Erlenmeyer flasks containing 50 ml of medium (pH 5.5) and initial cell concentration (1.0 g/l) of *S. stipitis*. They were maintained in a rotator shaker at 200 rpm and 30 °C for 96 h.

### Analytical methods

Xylose, glucose and ethanol concentrations were analyzed by HPLC (Schimadzu LC-10 AD (Kyoto, Japan) with column equipped with BIO-RAD Aminex HPX-87H (300 × 7.8 mm) coupled to a detector of refractive index RID-6A, with eluent 0.01 N sulfuric acid at a flow rate of 0.6 ml min^−1^, column temperature of 45 °C and injected volume of 20 μl. Before the passing of samples with HPLC, the samples were filtered through Sep Pak C18 filter. The standard chemicals were purchased from Sigma Aldrich (St. Louis, MO).

The determination of furfural and hydroxymethylfurfural (HMF) was measured by HPLC in the same chromatograph using a HP-RP18 column (200 × 4.6 mm), coupled to an UV detector SPD-10A UV–VIS, eluting with acetonitrile/water (1:8) with 1 % acetic acid with a flow rate of 0.8 ml/min, wavelength of 276 nm, column temperature 25 °C and injected sample volume of 20 μl. Before the readings, the samples are filtered through membranes Minisart 0.22 μm (Sartorius AG, Goettingen, Germany).

The concentration of free cells was determined by turbidimetry using spectrophotometer (Beckman DU 640 B Fullerton, CA) at wavelength of 600 nm and correlated with the dry weight of cells (g/l) through a calibration curve:

The measurements were made on diluted cell suspensions, after centrifugation, washing and re-suspension of cells in distilled water.

## Results and discussion

**Fig. 1:**
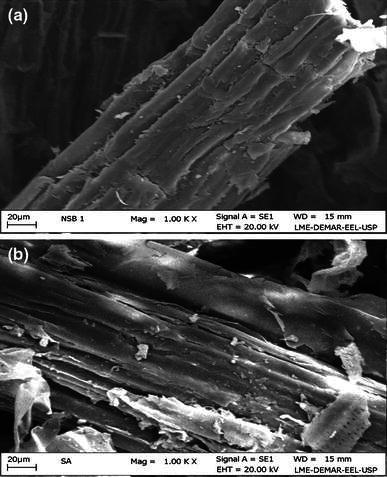
Scanning electron microscopy (SEM) of surface images of the sugarcane bagasse **a** native **b** dilute sulfuric acid pretreated

Dilute acid hydrolysis of sugarcane bagasse primarily depolymerizes the hemicellulose fraction of cell wall into monomeric constituents. During thermochemical reactions, sulfuric acid acts as catalyst, which at high temperatures (120–180 °C) for few minutes of residence time promote cleavage of *β*-1,4 xylosidic linkages present in hemicellulose and accompanying side chains into xylose and other byproducts (Alvira et al. 2010) leaving cellulose and lignin together but in fragile form. Figure 1 shows the scanning electron microscopy (SEM) of the native sugarcane bagasse and after pretreatment with dilute sulfuric acid. It is clearly revealed that sulfuric acid mechanistically acts on hemicellulose degradation and disturbing the surface morphology. The pretreated bagasse in now amenable to cellulase-mediated enzymatic action for its de-polymerisation into glucose.

**Fig. 2:**
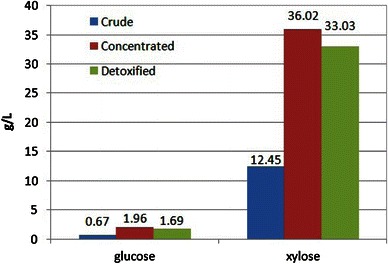
Sugars profile of sugarcane bagasse hemicellulosic hydrolysate

**Fig. 3:**
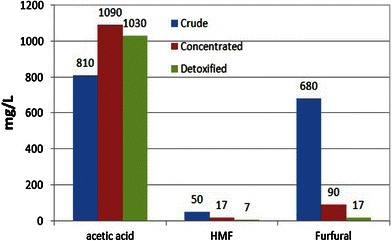
Inhibitors profile of sugarcane bagasse hemicellulosic hydrolysate

In addition to sugar monomers (xylose, arabinose, glucose, mannose, galactose), other compounds such as furfurals, 5-HMF, phenolics, weak acids and metals are also generated (Figs. 2, 3). Acetic acid is also released in hydrolysates due to the cleavage of acetyl chain linked to xylose backbone. Inhibitors like furfural and 5-HMF are originated from the sugar degradation during the hydrolysis process (Fonseca et al. 2011). Phenolics are generated in hydrolysates from lignin degradation. These compounds are known as inhibitors to the fermenting microorganisms and affect ethanol yields and productivities. Therefore, it is necessary to eliminate these inhibitors from the hydrolysates prior to microbial fermentation.

The hydrolysate revealed high-xylose concentrations, about 18 times more than glucose, indicating that under these hydrolysis conditions hemicellulose was more susceptible to hydrolysis than cellulose (Canilha et al. 2010). Fonseca (2009) reported similar values, 12.0 g/l of xylose and 0.41 g/l of glucose, from sugarcane bagasse hydrolysate, under the same hydrolysis conditions. The absence of arabinose in sugarcane bagasse hydrolysate can be attributed to the origin and type of sugarcane bagasse used in these experiments. This fact has also been observed previously in the report of Antunes et al. (2011), which did not find arabinose in the sugarcane bagasse acid hydrolysate employing the similar hydrolytic conditions.

The dilute acid hydrolysate was concentrated by vacuum evaporation which results approximately threefold hike the concentration of the associated components. Figure 2 shows the profile of xylose and glucose in native, vacuum concentrated and sequentially detoxified sugarcane bagasse hemicellulosic hydrolysates. Glucose concentration was determined to be 0.67, 1.96 and 1.69 g/l in natural, vacuum concentrated and detoxified hydrolysates, respectively. Xylose concentrations were 12.45, 36.02 and 33.03 g/l in natural, vacuum concentrated and detoxified hydrolysates, respectively. The goal of the concentration step was to increase the monomeric sugar levels in the hydrolysate to provide the considerable sugar levels to fermenting microorganism. However, vacuum concentration also increased inhibitor concentrations. Detoxification steps efficiently removed inhibitory components from the hydrolysates. Figure 3 presents the profile of inhibitors concentrations of detoxified hydrolysate (1,030 mg/l acetic acid, 7 mg/l.5-HMF and 17 mg/l furfural). These values were lower than those determined in the concentrated hydrolysate (1,090 mg/l acetic acid, 17 mg/l.5-HMF and 90 mg/l furfural). Calcium oxide mediated neutralization of acid hydrolysate followed by charcoal adsorption significantly removed acetic acid and furans from the lignocellulose hydrolysates (Alves et al. 1998). Sugarcane bagasse hemicellulosic hydrolysate when treated with calcium oxide mediated neutralization and activated charcoal showed 38.7, 57 and 46.8 % reduction in furans, phenolics and acetic acid, respectively (Chandel et al. 2007).

We observed 81 % furfural and 61 % of HMF reductions after sequential detoxification of hydrolysates. Initially, the xylose/glucose ratio was approximately 18.4 in native hydrolysate which increased to 19.5 after detoxification. The glucose concentration (1.69 g/l) corresponds to 5.12 % of xylose. This low ratio is desirable since hexose presence can prejudice pentose metabolism due to catabolite repression (Young et al. 2010).

## Fermentation of hydrolysate

Presence of pentose and hexose sugars in hydrolysates is good to obtain the desired ethanol yields from *S. stipitis* after fermentation. Considering that yeasts require a variety of substances for the synthesis of cellular material and energy (such as carbon, oxygen, nitrogen, etc.), the presence of these nutrients is essential for a good performance of a microorganism in fermentation processes (Ferreira et al. 2011). Aiming to replace the conventional and expensive nitrogen sources such as yeast extract and peptone in fermentation processes, RBE was used as a nitrogen source in the sugarcane bagasse hemicellulosic hydrolysate to produce economic ethanol by the yeast *S. stipitis*.

**Fig. 4:**
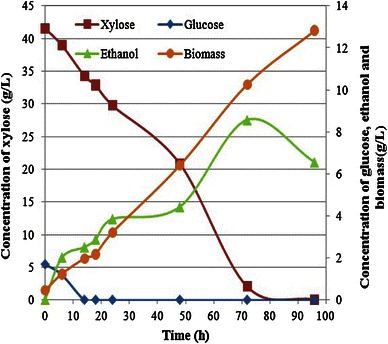
Fermentation of medium #3 (detoxified sugarcane bagasse hemicellulosic hydrolysate + rice bran extract + ammonium sulfate + calcium chloride) using *S. stipitis* NRRL Y-7124 at 30 °C and pH 5.0

Figure 4 shows the complete fermentation profile of medium #1 (sugarcane bagasse hemicellulosic hydrolysate + RBE + ammonium sulfate + calcium chloride) using *S. stipitis*. It was found that *S. stipitis* growth reached of 12.8 ± 0.4 g/l in 96 h, which was continued until the end of fermentation. Maximum ethanol (8.6 ± 0.1 g/l, yield 0.22 g/g and productivity 0.12 g/l h) was attained after 72 h of incubation and thereafter it showed a regular declination. It is noteworthy to mention here that there were no sugars available after 72 h, however, yeast growth was regular increasing with the downfall in ethanol concentration suggesting that microorganism started to consume ethanol as carbon source after the exhaustion of sugars in medium, which is a common feature in xylose fermenting yeasts (Chandel et al. 2007). Both the sugars (glucose and xylose) were simultaneously consumed by the microorganism. All the glucose was consumed by *S. stipitis* within the initial 12 h. Xylose consumption profile shows the regular downfall with the concomitant ethanol production and hikes in biomass production (Fig. 4). Similar growth and ethanol production pattern was also observed by (Mussatto and Roberto 2002) using the yeast *Candida guilliermondii* FTI 20037 grown in rice straw hemicellulosic hydrolysate.

Other two fermentation media #2 and #3 were also tested to compare with RBE performance. Table 1 summarizes the kinetic parameters of the fermentation of all three different fermentation media by *S. stipitis*. Fermentation medium #1 showed maximum ethanol production (8.6 g/l, yield 0.22 g/g) followed by medium #2 (8.1 g/l, yield 0.19 g/g) and medium #3 (7.4 g/l, yield 0.18 g/g). However, medium #3 showed maximum yeast cell mass production (yield 0.34 g/g, productivity 0.20 g/l h) followed by medium #2 (0.33 g/g, productivity 0.19 g/l h) and medium #1 (0.26 g/g, productivity 0.14 g/l h). Cell mass production pattern in all the three media formulation suggests the preference of yeast toward the medium #3, which is nutrient enriched medium followed by medium #2 and medium #1. In the medium #1, RBE with the main nitrogen source revealed maximum ethanol production with the minimum cell mass production. Fermentation medium composed of nitrogen sources i.e. peptone and/or yeast extract led better biomass production than ethanol production are not certainly of interest due to increasing the production cost.

**Table 1:** Ethanol production performance of *S. stipitis* NRRL Y-7124 using three different fermentation medium

Fermentation medium	Ethanol (g/l)	Y_X/S_	Y_P/S_	Q_p_	Q_x_	Q_s_
1	8.6 ± 0.1	0.26	0.22	0.12	0.14	0.55
2	8.1 ± 0.1	0.33	0.19	0.11	0.19	0.58
3	7.4 ± 0.2	0.34	0.18	0.10	0.20	0.58

Fermentation medium #1 (detoxified sugarcane bagasse acid hydrolysate + RBE + ammonium sulfate + calcium chloride), fermentation medium #2 (detoxified sugarcane bagasse acid hydrolysate + yeast extract + peptone) and fermentation medium #3 (detoxified sugarcane bagasse acid hydrolysate + yeast extract + peptone + malt extract) after 72 h of incubation

*Y*_*x/s*_ biomass yield and *Y*_*P/S*_ ethanol yield are in g/g and *Q*_*x*_ biomass productivity, *Q*_*p*_ ethanol productivity and *Q*_*s*_ sugar consumption rate in g/l h

RBE is a cheap nitrogen source which can be obtained following a simple method of autohydrolysis. According to Kahlon (2009), rice bran contains about 2.4 % of total nitrogen, whereas, the yeast extract and peptone have around 10.9 % (Vetec Quimica fina) and 12.5 % (Synth) of total nitrogen, respectively. Carbohydrates, fat and nitrogen are the basic components of rice bran constituting approximately 76.81 % of total fraction (Table 2). This study shows the improved ethanol production from SB hemicellulosic hydrolysate supplemented with RBE as nitrogen source by *S. stipitis.* Our results are promising when compared with previous studies carried out with different strains of *S. stipitis* for ethanol production under batch cultivation conditions using costly nitrogen sources. For example, Ferreira et al. (2011) obtained an ethanol yield (*Y*_P/S_) of 0.19 g/g and a productivity of 0.13 g/l.h using the yeast *S. stipitis* UFMG-IMH 43.2 from SB hemicellulosic hydrolysate supplemented with 5.0 g/l of yeast extract. Canilha et al. (2010) obtained 6.1 g/l with the yield of 0.30 g/g and a productivity of 0.13 g/l.h using SB hemicellulosic hydrolysate supplemented with 3.0 g/l of yeast extract, 3.0 g/l of malt extract and 5.0 g/l of peptone.

**Table 2:** Rice bran composition (Jiamyangyuen et al. 2005)

Component	Composition (%)
Moisture	8.5
Protein	12.6
Fat	21.13
Crude fiber	5.59
Ash	8.97
Carbohydrate	43.12

Microorganisms require moderate nitrogen amount for their vegetative growth during fermentation. The potential of de-oiled rice bran was evaluated by Ravinder et al. (2006) for the protein enrichment (24.80 %) by *Aspergillus oryzae* MTCC 1846 under solid state fermentation. Canilha et al. (2005) investigated the potential of RBE on xylitol production by the yeast *Candida guilliermondii* FTI 20037 grown in wheat straw hemicellulosic hydrolysate. They observed that maximum production of xylitol was 24.17 g/l with the yield (*Y*p/s) of 0.49 g/g and a productivity of 0.34 g/l.h after 72 h using 1.0 g/l of ammonium sulfate and 5.0 g/l of RBE as nitrogen source. Martinez and Santos (2012) reported 10 % v/v xylitol production from SB hemicellulosic hydrolysate supplemented with 2.5 % v/v of RBE as nitrogen source.

## Conclusion

In order to reduce the production cost of 2G ethanol, the use of RBE proves to be a cost effective and efficient alternative to the conventional nitrogen sources such as yeast extract and peptone in fermentation processes. The present study demonstrated that the ethanol productivity and yields from sugarcane bagasse hemicellulosic hydrolysates by *S. stipitis* were superior using RBE as nitrogen source than other nitrogen supplements. Rice bran is a renewable feedstock available in huge amount in the world and its extract has shown a potential to replace the costly organic and inorganic nitrogen sources for the economic ethanol production from second generation feedstocks in biorefineries. However, further studies are required first to optimize the appropriate conditions for RBE recovery adopting autohydrolysis based methods and second the use of suitable amount of RBE in fermentation process as nitrogen sources.

## References

[CR1] Agbogbo FK, Coward-Kelly G (2008). Cellulosic ethanol production using the naturally occurring xylose-fermenting yeast, *Pichia stipitis*. Biotechnol Lett.

[CR2] Alves LA, Felipe MGA, Almeida e Silva JB, Silva SS, Prata AMR (1998). Pretreatment of sugarcane bagasse hemicellulose hydrolisate for xylitol production by Candida guilliermondii. Appl Biochem Biotech.

[CR3] Alvira P, Tomás-pejó E, Ballesteros M, Negro MJ (2010). Pretreatment technologies for an efficient bioethanol production process based on enzymatic hydrolysis: a review. Bioresour Technol.

[CR4] Ananda N, Vadlani PV, Madl RL (2011). Rice Bran is an effective substitute for yeast extract in ethanol fermentation. J Biobased Mater Bioener.

[CR5] Antunes FAF, Milessi TSS, Chandel AK, Silva SS (2011) Characterization of sugarcane bagasse hemicellulosic hydrolysate after detoxification with overliming and activated charcoal. In: 20th European Biomass Conference and Exhibition, pp 1603–1606

[CR6] Arruda PV (2007) Efeito do glicerol na bioconversão de xilose em xilitol pela levedura *Candida guilliermondii* FTI 20037. Master Dissertation, Universidade de São Paulo

[CR7] Canilha L, Carvalho W, Almeida e Silva JB (2005). Influence of médium composition on xylitol bioproduction from wheat straw hemicellulosic hydrolysate. World J Microb Biotechnol.

[CR8] Canilha L, Carvalho W, Felipe MGA, Almeida e Silva JB, Giulietti M (2010). Ethanol production from sugarcane bagasse hydrolysate using Pichia stipitis. Appl Biochem Biotech.

[CR10] Chandel AK, Kapoor RK, Singh A, Kuhad RC (2007). Detoxification of sugarcane bagasse hydrolysate improves ethanol production by *Candida shehatae* NCIM 3501. Bioresour Technol.

[CR11] Chandel AK, Silva SS, Carvalho W, Singh OV (2012). Sugarcane bagasse and leaves: foreseeable biomass of biofuel and bio-products. J Chem Technol Biotechnol.

[CR12] Dale BE, Ong RG (2012). Energy, wealth, and human development: why and how biomass pretreatment research must improve. Biotechnol Progr.

[CR13] Ferreira AD, Mussatto SI, Cadete RM, Rosa CA, Silva SS (2011). Ethanol production by a new pentose-fermenting yeast strain, *Scheffersomyces stipitis* UFMG-IMH 43.2, isolated from the Brazilian Forest. Yeast.

[CR14] Fonseca BG (2009) Destoxificação biológica de hidrolisado hemicelulósico de bagaço de Cana-de-açúcar empregando as leveduras *Issatchenkia occidentalis* e *Issatchenkia orientalis*. Master Dissertation, Universidade de São Paulo

[CR15] Fonseca BG, Moutta RO, Ferraz FO, Vieira ER, Nogueira AS, Baratella BF, Rodrigues LC, Hou-Rui Z, Silva SS (2011). Biological detoxification of different hemicellulosic hydrolysates using *Issatchenkia occidentalis* CCTCC M 206097 yeast. J Ind Microbiol Biotechnol.

[CR16] Gírio FM, Fonseca C, Carvalheiro F, Duarte LC, Marques S, Bogel-Lukasik R (2010). Hemicelluloses for fuel ethanol: a review. Bioresour Technol.

[CR1000] Jeffries TW, Jin YS (2004) Metabolic engineering for improved fermentation of pentoses by yeasts. Appl Microbiol Biotechnol 63:495–509 10.1007/s00253-003-1450-0 14595523

[CR1001] Jiamyangyuen S, Srijesdaruk V, Harper WJ (2005) Extraction of rice bran protein concentrate and its applicationin bread. Songklanakarin J Sci Technol 27:55–64

[CR17] Kahlon TS, Cho SS, Samuel P (2009). Rice bran: production, composition, functionality and food applications, physiological benefits. Fiber ingredients: food applications and health benefits.

[CR18] Kurtzman CP, Suzuki M (2010). Phylogenetic analysis of ascomycete yeasts that form coenzyme Q-9 and the proposal of the new genera *Babjeviella*, *Meyerozyma*, *Millerozyma*, *Priceomyces*, and *Scheffersomyces*. Mycoscience.

[CR19] Macrelli S, Mogensen J, Zacchi G (2012). Techno-economic evaluation of 2nd generation bioethanol production from sugar cane bagasse and leaves integrated with the sugar-based ethanol process. Biotechnol Biofuels.

[CR20] Martínez EA, Santos JAF (2012). Influence of the use of rice bran extract as a source of nutrients on xylitol production. Ciênc Tecnol Aliment.

[CR21] Milessi TSS, Chandel AK, Branco RF, Silva SS (2011). Effect of dissolved oxygen and inoculum concentration on xylose reductase production from *Candida guilliermondii* using sugarcane bagasse hemicellulosic hydrolysate. Food Nutr Sci.

[CR22] Moro JD, Rosa CS, Hoelzel SCS (2004). Composição centesimal e ação antioxidante do farelo de arroz e seus benefícios à saúde. Ciências da Saúde.

[CR23] Mussatto SI, Roberto IC (2002). Produção biotecnológica de xilitol a partir da palha de arroz. Biotecnologia Ciênc Desenvolv.

[CR24] Pessoa A, Mancilha IM, Sato S (1997). Acid hydrolysis of hemicellulose from sugarcane bagasse. Braz J Chem Eng.

[CR25] Ravinder R, Chandel AK, Rao LV, Ravindra P (2006). Optimization of protein enrichment of de-oiled rice bran by solid state fermentation using *Aspergillus oryzae* MTCC 1846. Int J Food Eng.

[CR26] Soccol CR, Vandenberghe LPS, Medeiros ABP, Karp SG, Buckeridge M, Ramos LP, Pitarelo AP, Ferreira-Leitão V, Gottschalk LMF, Ferrara MA, Bon EBS, Moraes LMP, Araújo JA, Torres FAG (2010). Bioethanol from lignocelluloses: status and perspectives in Brazil. Bioresour Technol.

[CR27] Young E, Lee S, Alper H (2010). Optimizing pentose utilization in yeast: the need for novel tools and approaches. Biotechnol Biofuels.

